# Gain-of-function of TRPM4 predisposes mice to psoriasiform dermatitis

**DOI:** 10.3389/fimmu.2022.1025499

**Published:** 2022-10-20

**Authors:** Daisuke Yamada, Simon Vu, Xuesong Wu, Zhenrui Shi, Desiree Morris, Joshua D. Bloomstein, Mindy Huynh, Jie Zheng, Samuel T. Hwang

**Affiliations:** ^1^ Department of Dermatology, University of California, Davis, Sacramento, CA, United States; ^2^ Department of Physiology and Membrane Biology, University of California, Davis, Davis, CA, United States; ^3^ Department of Dermatology, Sun Yat-sen Memorial Hospital, Sun Yat-sen University, Guangzhou, China; ^4^ Kirk Kerkorian School of Medicine at University of Nevada, Las Vegas, Las, Vegas, NV, United States

**Keywords:** TRPM4, keratinocyte, dendritic cell, psoriasis, imiquimod (IMQ), mouse model, progressive symmetric erythrokeratodermia, glibenclamide

## Abstract

Transient receptor potential melastatin 4 (TRPM4) is a Ca^2+^-activated, monovalent cation channel that is expressed in a wide range of cells. We previously reported two gain-of-function (GoF) mutations of TRPM4 as the cause of progressive symmetric erythrokeratodermia (PSEK), which shares similar clinical and histopathological features with psoriasis. Using CRISPR/Cas9 technology, we generated TRPM4^I1029M^ mice that have the equivalent mutation to one of the two genetic mutations found in human PSEK (equivalent to human TRPM4^I1033M^). Using this mutant mice, we examined the effects of TRPM4 GoF at the cellular and phenotypic levels to elucidate the pathological mechanisms underlying PSEK. In the absence of experimental stimulation, TRPM4^I1029M^ mice did not show a phenotype. When treated with imiquimod (IMQ), however, TRPM4^I1029M^ mice were predisposed to more severe psoriasiform dermatitis (PsD) than wild-type (WT), which was characterized by greater accumulation of CCR6-expressing γδ T cells and higher mRNA levels of *Il17a*. In TRPM4^I1029M^ mice, dendritic cells showed enhanced migration and keratinocytes exhibited increased proliferation. Moreover, a TRPM4 inhibitor, glibenclamide, ameliorated PsD in WT and TRPM4^I1029M^ mice. Our results indicate elevated TRPM4 activities boosted susceptibility to cutaneous stimuli, likely through elevation of membrane potential and alteration of downstream cellular signaling, resulting in enhanced inflammation. Our results further suggest a possible therapeutic application of TRPM4 inhibitors in psoriasis.

## Introduction

Transient receptor potential melastatin 4 (TRPM4) is a Ca^2+^-activated nonselective cation (CAN) channel involved in diverse physiological processes and implicated in several human hereditary diseases (1, 2). Increase in TRPM4 activity induced by elevating intracellular Ca^2+^ concentration is expected to depolarize the membrane potential and induce subsequent cellular response (3). Like most transient receptor potential (TRP) channels, TRPM4 is thought to be tetramer made of four subunits, each of which containing six transmembrane segments and intracellularly located N- and C-terminal. Recently, its cryo-EM structure was reported by four independent groups, showing a four-fold symmetrical complex (4–7). TRPM4 proteins are found in various cell types and determine important functions, including insulin secretion in pancreatic beta cells, cardiac excitability in ventricular cells, immune response in lymphocytes, dendritic cells (DCs), and mast cells as well as excitability and contractility in vascular smooth muscle cells (8–14). Several heterozygous mutations of TRPM4, leading to gain-of function (GoF) have been identified in familial heart conduction diseases (15, 16), signifying its importance in maintaining cellular homeostasis. Recently, two GoF mutations (p.I1033M and p.I1040T) in TRPM4 were found in patients with an autosomal dominant-inherited skin disease termed progressive symmetric erythrokeratodermia (PSEK). Both of the mutation sites are located in ion permeation pore region. The mutant channels were found to induce increased sensitivity to Ca^2+^ leading to elevation of resting membrane potential and keratinocyte proliferation (17). These new findings suggest TRPM4 may be important in the maintenance of skin homeostasis as well as response to inflammatory stimuli.

Abnormalities in the immune response are involved in many dermatoses, including potentially PSEK. TRPM4 affects the function of various types of immune cells. For example, TRPM4 has been demonstrated to regulate migration, but not the maturation, of DCs (13). Inhibition of *TRPM4* by siRNA in Jurkat T cells resulted in enhanced IL-2 production (18), and inhibition of *TRPM4* expression led to increased Ca^2+^ influx and oscillatory levels in Th2 cells, but decreased Ca^2+^ influx and oscillations in Th1 cells. Inhibition of *TRPM4* expression also significantly altered T cell cytokine production and motility (12). Other studies indicate that TRPM4 affected the function of monocytes, macrophages (19) and mast cells (14, 20), but does not alter intracellular Ca^2+^ mobilization in neutrophils (19).

*In vivo* roles of TRPM4 have been explored in several experimental animal models using TRPM4 knockout (TRPM4^-/-^) mice. TRPM4^-/-^ mice have shown increased mortality in a model of sepsis induced by cecal ligation and puncture (19), as well as more severe IgE-mediated acute cutaneous anaphylactic response (14). Additionally, they showed reduced axonal and neuronal degeneration and attenuated clinical disease scores in mice models of experimental autoimmune encephalomyelitis (EAE), but this occurred without altering EAE-relevant immune function (21). While these studies highlighted the role of TRPM4 in maintaining cellular homeostasis including the immune system, it remains unclear how TRPM4 GoF mutants lead to PSEK and other human diseases. To address this question, we generated TRPM4 GoF mice using the CRISPR/Cas9 method which have the equivalent mutation to one of the two genetic mutations found in human PSEK (equivalent to human TRPM4^I1033M^). The mutation site is located in ion permeation pore region (**Supplementary Figure 1**). The TRPM4 GoF mice did not show any skin phenotype in the absence of stimulation, however, the results of our *in vitro* experiments indicated that this mutation affects the membrane potential and proliferation of keratinocytes, suggesting that cells harboring GoF mutant TRPM4 channels might be more susceptible to disease-causing environmental stimuli. Considering that the skin manifestations and histopathological findings of PSEK are similar to those of psoriasis (17) as manifested by thickened, red scaly plaques, we employed an imiquimod (IMQ)-induced PsD model. When stimulated by IMQ, TRPM4 GoF mice showed enhanced cutaneous inflammation. Both GoF mice and wild-type (WT) littermates showed reduction of IMQ-induced dermatitis when treated with glibenclamide, a TRPM4 inhibitor. Thus, TRPM4 plays a critical role in regulating psoriasis-like features in mice, which possibly explains the resemblance of the skin lesions in PSEK patients to human psoriasis and may point to TRPM4 as a relevant target in psoriasis.

## Materials and methods

### Generation of TRPM4 mutant (I1029M) mice (C57BL/6J background)

To generate TRPM4 I1029M mice, we obtained purified Cas9, a TRPM4-targeting guide RNA (gRNA; 5’-CAGGTTGAGCAACAGGATATTGG-3’) that targets Cas9 to a site near the I1029 codon, and donor oligos for HDR resulting in p.I1029M (c.C3087G) mutations (HDR donor sequence; 5’-CCCAGTATGCCAACTGGCTGGTGGTGTTGCTCCTTATCGTCTTCTTGCTGGTGGC CAATATGCTGTTGCTCAACCTGCTCATCGCCATGTTCAGGTGTGCCT-3’). Purified Cas9, TRPM4-targeting gRNA, and HDR oligos were procured from Integrated DNA Technologies. All three products were injected into C57BL/6J zygotes at the University of California, Davis mouse biology program. C57BL/6J mice were purchased from Jackson laboratories. The resulting pups were genotyped by amplifying a 374 bp region flanking the I1029M site and subsequent Sanger sequencing. Heterozygous mice from the founder line were then intercrossed to produce litters with WT, heterozygous, and homozygous animals. Mice were maintained under specific pathogen free conditions throughout this study. For the genotyping of the mice, we used RT-qPCR of mutant *TRPM4*, i.e., homozygous mouse expressed twice as high mutant *TRPM4* as heterozygous mice whereas no mutant *TRPM4* was detected in WT mouse. All animal experiments were performed under protocols (#20960) approved by the Institutional Animal Care and Use Committee at the University of California, Davis.

### Primary keratinocyte isolation and culture

Adult mouse keratinocytes were cultured following the procedure described by Li (22). Briefly, the tail skin from an adult mouse was digested in dispase digestion buffer, which contains 4 mg/ml dispase II in supplemented KC growth medium (KC basal medium with 0.06 mM CaCl2, Defined Growth Supplement, antibiotics) overnight 4°C. Epidermal sheet was removed, digested in trypsin-based digestion solution. Then cells were filtered and seeded in supplemented KC growth medium in culture dishes pre-coated with a collagen-based coating material. For WST-1 assay, cells were seeded at 1.0x10^5^ cells/well in a 96-well plate. After 24 hours of incubation, medium was changed and thereafter WST-1 (Sigma-Aldrich, St. Louis, MO) was added to each well. For cell counting, 1.0x10^5^ cells/well were seeded in a 24-well plate. After 4 days of incubation, cell numbers were counted using a hemocytometer.

### Electrophysiology

Recordings were performed using a HEKA EPC10 amplifier with PatchMaster software. The bath solution consisted of 140 mM NaCl, 5 mM KCl, 1 mM CaCl_2_, 1 mM MgCl_2_, 10 mM HEPES, 10 mM Glucose, pH 7.4; the pipette solution consisted of 150 mM K-Asp, 5 mM KCl, 10 mM HEPES, 10 µM PIP2, 2 mM EGTA, 1.8 mM CaCl_2_, pH 7.4 (resulting in 10 µM free Ca^2+^, calculated by MAXCHELATOR program, http://maxchelator.stanford.edu). Patch pipettes were fashioned from borosilicate glass and fire polished within the range of 3-5 MΩ. All recordings were performed at room temperature (~22°C). Membrane potential was recorded in the whole-cell patch configuration. The liquid junction potentials were tested experimentally and adjusted accordingly.

### Cell cycle assay

Cell cycle assay was performed following the procedure described by Kim (23). Briefly, primary keratinocytes isolated from adult mouse tail were fixed in ice cold 70% ethanol. Then, the cells were stained with anti-Ki-67 antibody (Cat# 151211, BioLegend) and propidium iodide containing RNase (Cat# 4087, Cell Signaling Technologies).

### IMQ-induced PsD model

IMQ-induced PsD model was described previously (24). 50 mg of 5% IMQ (Aldara; 3M Pharmaceuticals) in total was applied once daily to both sides of both ears for 5 consecutive days. Vanicream (Pharmaceutical Specialties, Cleveland, GA, USA) was applied on ears of WT mice as a vehicle control.

### Scoring severity of skin inflammation

To score the severity of inflammation of the ear skin, an objective scoring system was used as described before (24). Briefly, erythema, scaling, and thickening were scored independently on a scale from 0 to 4: 0, none; 1, slight; 2, moderate; 3, marked; 4, very marked. We calculated the cumulative score (erythema plus scaling plus thickening) as psoriasis severity index (PSI) score (scale 0–12). Ear skin thickness was measured with a thickness gauge (Peacock, Ozaki, MFG.CO., LTD, Japan) having 0.01 mm accuracy.

### Histopathological analysis and immunohistochemistry

Formaldehyde-fixed, paraffin-embedded ear skin samples were stained with H&E using standard procedures. Images were acquired using a Nikon Optiphot 2 microscope (Nikon, Tokyo, Japan). Epidermal thickness was measured with a computer-assisted image analysis software. For immunohistochemical analysis, skin sections were incubated with primary antibodies against murine p-STAT3, followed by the appropriate secondary antibodies. Rinsed sections were counterstained with hematoxylin.

### DNFB-induced contact hypersensitivity

One day after shaving the back hair with electrical clippers, the back skin was treated with 50 µl of 0.5% DNFB (1-fluoro-2,4-dinitrobenzene) (Sigma-Aldrich, St. Louis, MO) in a 4:1 mixture of acetone and olive oil. Five days later, the mice were challenged with 40 µl 0.2% DNFB. WT mice without sensitization served as a control group. Ear thickness was evaluated before challenge and after challenge (24 hours, 48 hours, and 72 hours).

### Croton oil-induced irritant contact dermatitis

25 μl of 2% croton oil (in a 4:1 mixture of acetone and olive oil) was applied to the dorsal side of the ear. Ear thickness was evaluated 2, 4, 6, and 24 hours after challenge. WT mice applied with 4:1 mixture of acetone and olive oil served as a vehicle control group.

### Flow cytometry

Anti–mouse γδ-TCR (clone GL3), CD3 (17A2), CD45 (30-F11), CCR6 (29-2L17), CD11b (M1/70), CD11c (N418), and Ly6G (IA8) antibodies were from BioLegend (San Diego, CA). Anti-mouse IL-17A (eBio17B7) antibody was from eBioscience (San Diego, CA). Flow cytometry was performed and analyzed using an Accuri C6 (BD Biosciences, San Jose, CA). After recovery of mouse ears, the ears were cut into small pieces with a blade. To obtain cell suspensions, small pieces of ear skin were digested with Liberase TM (Roche, Mannheim, Germany) and DNase I (Sigma-Aldrich, St. Louis, MO) with addition of 5% fetal bovine serum for 90 minutes before passing tissue through a 100-μm cell strainer. Anti-mouse CD16/32 (BD, San Jose, CA) were added to cells prior to staining to block binding to Fc receptors. Flow gating for CCR6^+^ γδ-low T cells was performed using a gating strategy described in detail in our previous reports (25, 26). Representative flow gating for the population is shown in **Supplementary Figure 2**. Intracellular staining for IL-17A was done after incubating cells for 5 hours with cell stimulation cocktail plus protein transport inhibitors (eBioscience 00-4975-93).

### Quantitative real-time PCR

Total RNA of mouse ear skin was extracted using a RNeasy Fibrous Tissue Kit (Qiagen, Hilden, Germany). Quantitative real-time PCR was performed using CFX connect Real-Time PCR Detection System (Bio-Rad, Hercules, CA). Predesigned primers were obtained from Integrated DNA Technologies, Inc (Skokie, IL).

### DC migration assay

We applied 100μl of 1% FITC (Sigma-Aldrich, St. Louis, MO) solution in acetone and dibutyl phthalate (1:1) on mouse ears to label phagocytic antigen presentation cells, such as dermal and epidermal DCs, and quantified their migration from skin to regional LN. Six hours after the FITC application, we collected the cervical LNs and quantified the FITC^+^ DCs by flow cytometry.

### Glibenclamide treatment

We administered 10 µg glibenclamide daily by intra-peritoneal injections to all mice in the treatment group (21). We daily dissolved 25 mg glibenclamide (Sigma-Aldrich, St. Louis, MO) in 5 ml DMSO and diluted 200 µl of this solution in 9.8 ml PBS. Mice received 100 µl of this solution or 2% DMSO in PBS as vehicle control.

### Statistical analysis

All data are shown as mean ± SEM. Data were analyzed using GraphPad Prism version 8 (GraphPad Software, San Diego, CA). A two-sided unpaired Student’s t-test was used to compare two groups, and one-way analysis of variance (27) with Tukey’s *post hoc* test was used for multiple comparisons unless otherwise indicated. A p-value less than 0.05 was considered statistically significant.

## Results

### TRPM4 I1029M mutant mice show minimum phenotype without stimulation

**Figure 1:**
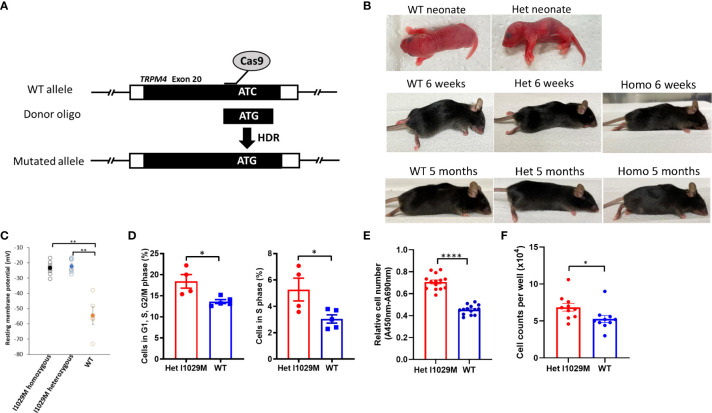
Het I1029M mice do not show phenotype without stimulation, however keratinocytes derived from the mutant mice are more proliferative. **(A)** Schematic diagram showing the site-directed mutation of ATC, encoding I1029, to ATG, encoding an M, using CRISPR/Cas9 technology. **(B)** Representative images of mice at neonate, 6-weeks, and 5-month. **(C)** Primary keratinocytes were isolated from the skin of I1029M homozygous, heterozygous, or WT mouse. Resting membrane potential was measured using patch-clamp technique with intracellular Ca^2+^ concentration of 100 μM (n = 5-8). **(D)** Left: Percentages of cells in G1, S, and G2/M phase. Right: Percentages of cells in S phase (n = 4-5). **(E)** The relative cell numbers of WT mice or Het mice-derived keratinocytes, determined by WST-1 assay (n = 14). **(F)** Cell numbers of WT mice or Het mice-derived keratinocytes, determined by cell counting (n = 8-10). Viable cell number was evaluated using WST-1 assay (n = 14). The data are presented as mean ± SEM. Data are representative of two independent experiments. *p < 0.05, **p < 0.01, ****p < 0.0001 by using one-way ANOVA with Tukey’s test in multiple comparison, Student’s T test in two group comparison.

To examine if mice harboring the human PSEK mutation can spontaneously exhibit a similar skin phenotype, we generated TRPM4 I1029M mutant mice using CRISPR/Cas9 methodology (**Figure 1A**; see Materials and Methods). This mutation is equivalent to human I1033M mutation. Briefly, purified Cas9, TRPM4-targeting guide RNA (gRNA), and homology directed repair (HDR) oligos were injected into C57BL/6J zygotes. The resulting pups were genotyped by amplifying a 374 bp region flanking the I1029M site and subsequent Sanger sequencing. Since clinical manifestations of PSEK are erythematous hyperkeratotic plaques at hands, foots, or periorificial sites, we observed those sites carefully. TRPM4 I1029M GoF mice, housed under standard specific-pathogen free conditions, did not show an obvious skin phenotype throughout the first 5 months of their lives (**Figure 1B**). Because other TRPM4 mutations in humans have been associated with familial heart block (9), we performed electrocardiograms in our mutant mice and found no abnormalities compared to WT siblings (data not shown). This is consistent with clinical observations from PSEK patients who do not have cardiac abnormalities (17).

### TRPM4 I1029M mutant keratinocytes exhibit an elevated resting membrane potential

Since we did not find obvious skin changes in TRPM4 GoF mice *in vivo*, we next asked if cells harboring this mutation show any changes compared to WT cells *in vitro*. Previously, we reported that the two TRPM4 missense mutations (I1033M, I1040T; human) exhibited an elevated resting membrane potential in HEK293 cells (17). To determine if this phenomenon occurs in TRPM4 mutant cells *in vitro*, we compared the resting membrane potential in isolated primary keratinocytes from TRPM4 I1029M mutant (and WT) mice. Indeed, the resting membrane potential was elevated in homozygous and heterozygous keratinocytes compared to WT (-23.6 ± 4.3 mV, -22.2 ± 3.7 mV, -54.6 ± 5.8 mV; homozygous, heterozygous, and WT, respectively) (**Figure 1C**). These results confirmed that TRPM4 activity is indeed enhanced (as expected) with the I1029M mutation. Notably, there was no obvious difference between homozygous and heterozygous mutation with regard to the resting membrane potential. Thus, despite similarities in baseline skin characteristics in TRPM4 mutant and WT mice *in vivo*, keratinocytes from the GoF mutant show elevated resting membrane potential.

### TRPM4 I1029M mutant keratinocytes exhibit enhanced proliferation

Since one of the major histological characteristics of PSEK is epidermal hyperplasia, we next asked whether TRPM4 I1029M mutation alters keratinocyte proliferation. In the electrophysiological study, we observed no substantial difference in resting membrane potential between homozygous and heterozygous keratinocytes (**Figure 1C**). This suggested that a single heterozygous mutation was enough to alter cellular physiology and was consistent with the autosomal dominant genetic inheritance of PSEK. While homozygous mutant mice were generated along with heterozygous TRPM4 GoF mutant mice, our *in vivo* experiments focused on heterozygous mice (Het I1029M) in order to replicate the human PSEK disease, which is only known to occur in the heterozygous state. Comparing Het I1029M and WT keratinocytes, we found that I1029M keratinocytes were more likely to be in G1, S, G2/M phase or S phase (**Figure 1D**). WST-1 assay was performed to examine the number of viable cells after 24 hours of incubation with equal numbers of either Het mice-derived or WT mice-derived keratinocytes. The relative cell number of Het mice-derived keratinocytes was significantly higher than those of WT mice-derived keratinocytes (**Figure 1E**). We performed cell counting, which also confirmed Het keratinocytes were more proliferative (**Figure 1F**).

Thus, these results show that the Het I1029M mutation stimulated keratinocytes proliferation *in vitro*.

### Het I1029M mice exhibit enhanced susceptibility to IMQ-induced psoriasiform dermatitis

**Figure 2:**
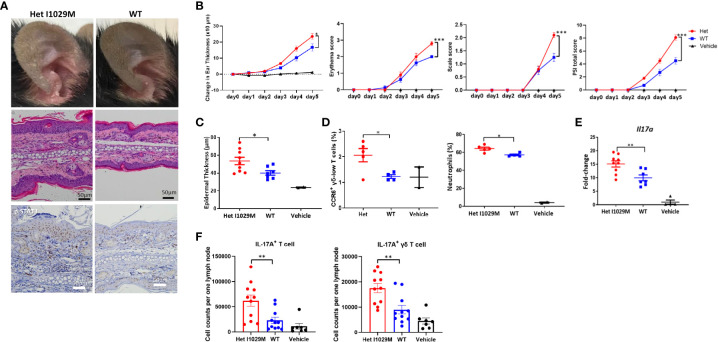
Het I1029M mice have enhanced susceptibility to PsD in response to IMQ treatment with more accumulation of CCR6 expressing γδ-low T cells and higher expression levels of *Il17a.* IMQ was applied to the ears of Het I1029M or WT mice for five consecutive days. **(A)** Representative photographs, images of H&E section (scale bars, 50 μm), and images of immunohistochemistry for p-STAT3. **(B)** Changes in ear thickness, erythema score, scale score, and total PSI score (n = 8-10, vehicle; n = 4). **(C)** Histological analysis of epidermal thickness (n = 8-10, vehicle; n = 2). **(D)** Left: proportions of CCR6^+^ γδ-low T cells among CD45^+^ cells, right: proportions of neutrophils among CD45^+^ cells in the lesional skin, determined by flow cytometry (n = 4-5, vehicle; n =2). **(E)** *Il17a* expression levels in the ear skin (n = 8-10, vehicle; n =4). **(F)** Cell from the regional lymph node were collected and evaluated by flow cytometry (n = 11). Vanicream was applied on ears of WT mice as a vehicle control (vehicle; n = 7). The data are presented as mean ± SEM. Data are representative of two independent experiments. *p < 0.05, **p < 0.01, ***p < 0.001 by using Student’s t-test.

Although Het I1029M mice did not display any skin phenotype in the absence of stimulation (**Figure 1** B), the results of our *in vitro* experiments indicate that this mutation affects the membrane potentials and proliferation of keratinocytes (**Figures 1C–F**), suggesting that cells harboring GoF mutant TRPM4 channels might be more susceptible to disease-causing environmental stimuli. The skin manifestations and histopathological findings of PSEK are similar to those of psoriasis (17). Thus, to determine if there is a difference between Het I1029M and WT mice in response to stimulation, we used an IMQ-induced PsD model. As a vehicle control group, we applied Vanicream to WT mice. To see if the application of Vanicream alone can cause any difference between WT and Het I1029M mice, we compared WT and Het I1029M with applying Vanicream only and there was no difference between WT and Het I1029M (**Supplementary Figure 3**). Firstly, to determine if TRPM4 is expressed in psoriasis skin, we analyzed a publicly available RNA sequencing dataset and found that TRPM4 was expressed in both psoriasis skin lesions and healthy skin controls (Gene Expression Omnibus GSE117405). Het I1029M mice showed more severe inflammation that was characterized by increased ear thickening, erythema, and scale (**Figures 2A, B**). Changes in ear thickness and clinical score (as assessed by psoriasis severity index score, PSI score) were larger in Het I1029M mice versus their WT littermates (**Figure 2B**). Consistent with the observation that Het I1029M keratinocytes were more proliferative (**Figure 1D–F**), epidermal thickness was greater in Het I1029M mice than WT controls (**Figure 2C**). Interestingly, other parameters such as erythema and scale that reflect inflammation rather than keratinocyte proliferation also showed significant differences (**Figure 2B**), suggesting that this mutation could affect other aspects of inflammation in this mouse model.

### Het I1029M mice do not show altered phenotype in croton oil-induced irritant contact dermatitis or DNFB-induced allergic contact dermatitis

We next asked if TRPM4 GoF mice have susceptibility to other stimuli using two other dermatitis models, i.e., croton oil-induced irritant contact dermatitis and di-nitro-fluoro-benzene (DNFB)-induced allergic contact dermatitis. However, Het I1029M mice did not show altered phenotype in these contact dermatitis models (**Supplementary Figures 4**, **5**). In the croton oil-induced model, WT mice were treated with a 4:1 mixture of acetone and olive oil and served as a vehicle control group. In the DNFB-induced model, WT mice which received DNFB challenge only served as a control group. The results indicate that the effect of TRPM4 GoF had relatively specific functional effects in the PsD-related IMQ model compared to the other two models.

### Enhanced inflammation observed in Het I1029M mice is characterized by accumulation of CCR6-expressing γδ-low T cells and higher expression levels of *Il17a*

We next sought to characterize the immunological features of the enhanced skin inflammation observed in Het I1029M mice. One day following the last application of IMQ (day 5), mice were sacrificed and cell suspensions from the ears were prepared. We have previously shown that CC chemokine receptor-6 positive (CCR6^+^) γδ-low T cells are one of the dominant sources of IL-17A and play an important role in IMQ-induced PsD (28–30). We first evaluated this cell subset by flow cytometry. Het I1029M mice showed an increased proportion of CCR6^+^ γδ-low T cells as well as neutrophils among CD45^+^ cells (**Figure 2D**). We next evaluated mRNA expression levels from mouse ears. mRNA expression levels of *Il17a* were greater in Het I1029M mice than WT (**Figure 2E**). This was consistent with the IHC staining of p-STAT3 where Het I1029M mice showed higher expression of p-STAT3 (**Figure 2A**), which is a downstream signaling of IL-17A and plays important roles in psoriatic keratinocytes (31). mRNA expression levels of other cytokines such as *Il6* did not show a difference (data not shown). We next asked if IL-17A^+^ T cells in the regional lymph node (LN) were increased in Het I1029M mice. IMQ was applied on the mouse ears for 2 days, 24 hours after the second IMQ application, mice were sacrificed and cell suspension from cervical LNs was prepared. Indeed, greater numbers of IL-17A^+^ CD3^+^ T cell or IL-17A^+^ γδ T cells were observed in Het I1029M mice (**Figure 2F**). LN cells from non-stimulated WT or Het I1029M mice had similar rate of IL-17A^+^ γδ T cells (Supplementary **Figure 6**). Collectively, at the immunological level, Het I1029M mice displayed augmented skin inflammation mainly characterized by enhanced Th17 response compared to WT counterparts following application of IMQ.

### Enhanced accumulation of DC in Het I1029M mice

**Figure 3:**
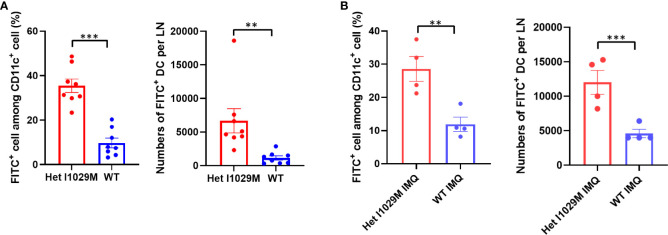
Enhanced accumulation of DCs is observed in Het I1029M mice. **(A)**100μl of 1% FITC solution was applied on mouse ears. Six hours after the FITC application, cervical LNs were collected. Left: increased FITC^+^ rate among CD11c^+^ cells, right: increased numbers of FITC^+^ DCs were observed in Het I1029M mice compared to WT (n = 8). **(B)** DC migration assay was conducted under IMQ applied condition. Left: increased FITC^+^ rate among CD11c^+^ cells, right: increased numbers of FITC^+^ DCs were observed in Het I1029M mice compared to WT (n = 4). Data are presented as mean ± SEM and are representative of two independent experiments. **p < 0.01, ***p < 0.001 by using Student’s T test.

We next sought to identify a functional explanation for the enhanced dermatitis in Het I1029M mice. Since TRPM4 contributes to efficient migration of DC (13), we hypothesized that DC migration is accelerated in Het I1029M mice. To test this idea, we applied 1% FITC solution on mouse ears to both label phagocytic antigen presentation cells, such as dermal and epidermal DC, to measure the efficiency of their migration from skin to regional LN. Six hours after the FITC application, we collected the cervical LNs and determined the FITC^+^ DC. Indeed, significantly increased numbers of FITC^+^ DC were observed in Het I1029M mice (**Figure 3A**), indicating that DC migration is enhanced in Het I1029M mice compared to WT mice. Moreover, this increased DC migration in Het I1029M mice was also observed under the skin was stimulated with IMQ (**Figure 3B**). Because DCs are one of the key players in PsD, it is possible that this increased migration of DCs contributes to the more severe inflammation observed in the mutant mice.

### Glibenclamide, a TRPM4 inhibitor, attenuates IMQ-induced PsD in Het I1029M and WT mice

**Figure 4:**
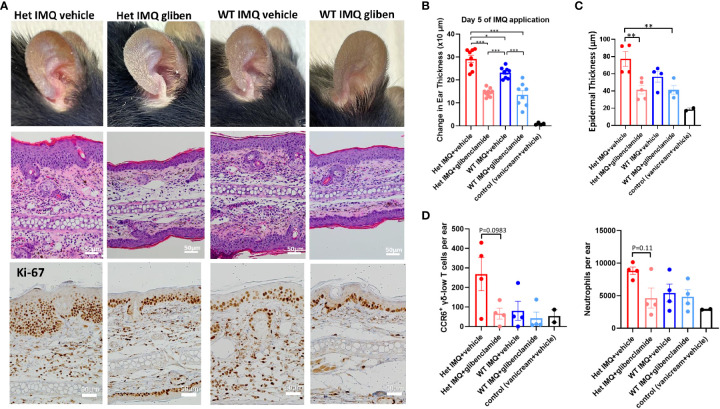
Glibenclamide, a TRPM4 inhibitor, attenuates IMQ-induced PsD in Het I1029M and WT mice. Het I1029M mice or WT mice received daily imiquimod application and daily glibenclamide i.p. injection for 5 days. **(A)** Representative photographs, histology images, and immunohistochemical images for Ki-67. **(B)** Changes in ear thickness (n = 8, vehicle; n = 4). **(C)** Histological analysis of epidermal thickness (n = 4, vehicle; n = 2). **(D)** Left: numbers of CCR6^+^ γδ-low T cells per ear, right: numbers of neutrophils per ear in the lesional skin (n = 4, vehicle; n = 2), determined by flow cytometry. The data are presented as mean ± SEM. Data are representative of two independent experiments. *p < 0.05, **p < 0.01, ***<0.001 by using one-way ANOVA with Tukey’s test.

**Figure 5:**
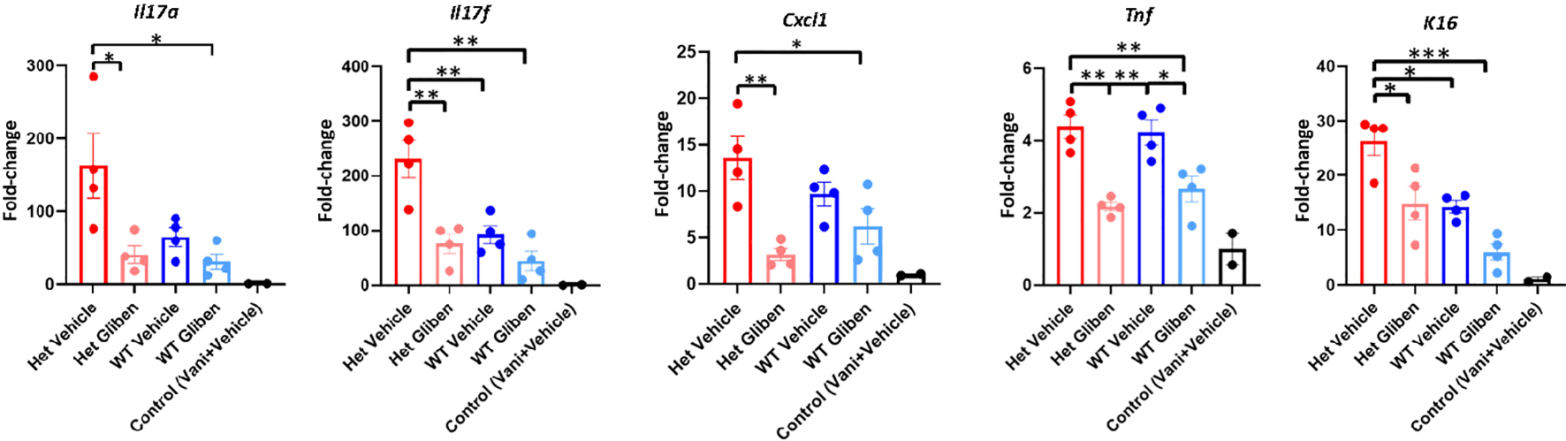
Glibenclamide ameliorates proinflammatory cytokine expressions. Het I1029M mice or WT mice received daily imiquimod application and daily glibenclamide i.p. injection for 5 days. RNA was extracted from mice ear skin. RT-qPCR was performed for *Il17a, Il17f, Cxcl1, Tnf, and K16.* The data are presented as mean ± SEM (n = 4, control; n = 2). Data are representative of two independent experiments. *p < 0.05, **p < 0.01, ***<0.001 by using one-way ANOVA with Tukey’s test.

**Figure 6:**
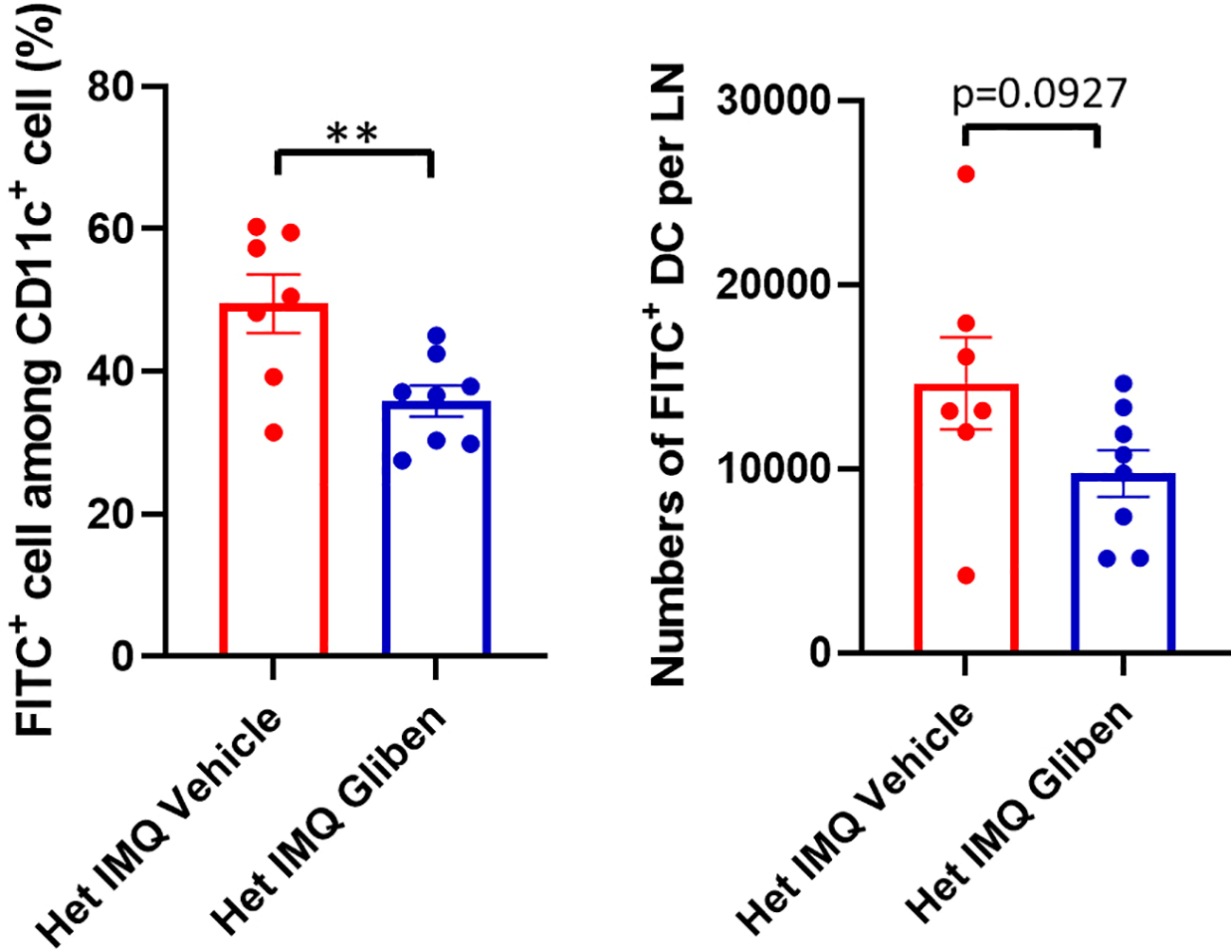
DC migration was attenuated by glibenclamide treatment. Glibenclamide was intraperitoneally injected, then IMQ was applied on mice ear. On the next day of the IMQ application, 1% FITC solution was applied on mice ear. Six hours after FITC application, the regional LN was collected, and evaluated by flow cytometry. Left: FITC^+^ rate among CD11c^+^ cells, right: numbers of FITC^+^ DCs. Data are presented as mean ± SEM (n = 7-8) and are representative of two independent experiments. **p < 0.01 by using Student’s T test.

Since TRPM4 GoF predisposes mice to more active PsD, we predicted that a TRPM4 inhibitor may ameliorate PsD. Glibenclamide, a classic diabetes drug, is known to also act as an inhibitor of TRPM4 (21). Het I1029M and WT mice received daily IMQ application and glibenclamide i.p. injection for 5 days. Indeed, glibenclamide ameliorated IMQ-induced PsD clinically and histologically in both groups of mice (**Figures 4A–C**). Clinical evaluation represented by the changes in ear thickness revealed that glibenclamide significantly ameliorated IMQ-induced PsD both in WT and Het I1029M mice (**Figure 4B**). In addition, glibenclamide treatment reduced the epidermal thickness in Het I1029M mice. Flow cytometry of mouse ear showed that the numbers of CCR6^+^ γδ-low T cells and neutrophils tended (although not reaching statistical significance) to be reduced by glibenclamide in Het I1029M mice (**Figure 4D**). Furthermore, mRNA levels of *Tnf* were reduced by glibenclamide both in WT and Het I1029M mice, and *Il17a*, *Il17f*, *Cxcl1*, and *K16* were reduced by glibenclamide in Het I1029M mice. (**Figure 5**). Lastly, we sought to investigate if DC migration is inhibited by glibenclamide. Glibenclamide was intraperitoneally injected, then IMQ was applied on mice ear. On the next day of the IMQ application, 1% FITC solution was applied on mice ear. Six hours after FITC application, the regional LN was collected and evaluated by flow cytometry. Indeed, DC migration was inhibited by glibenclamide (**Figure 6**). Thus, a known inhibitor of TRPM4 ameliorates PsD in TRPM4 mutant as well as WT mice, suggesting that TRPM4 may have a regulatory role in PsD and possibly introducing this protein as a therapeutic target in psoriasis.

## Discussion

Recently, we and our collaborators reported two TRPM4 GoF mutations that we suggest are the genetic basis of PSEK (17). Though PSEK is a genetic disease, it is clinically characterized by keratotic erythema that resembles the ones observed in inflammatory skin conditions such as psoriasis (32). Therefore, we hypothesized TRPM4 was involved in not only PSEK but could potentially play a regulatory role in other inflammatory skin diseases. To investigate the role of TRPM4 in the physiology and pathophysiology of dermatitis and PSEK, we generated TRPM4 GoF mutant mice (I1029M) that have an equivalent mutation to human PSEK.

Since PSEK is a disease that develops spontaneously during early childhood without known triggers or stimulation, we carefully observed the mutant mice from birth. Interestingly, our mutant mice did not show an obvious skin phenotype (**Figure 1B**) despite our observation that Het I1029M keratinocytes showed increased proliferation (**Figures 1D–F**). We speculate that compensatory mechanisms may inhibit the tendency of the mutant keratinocytes to undergo enhanced proliferation. Another important fact is that PSEK is a spontaneously resolving disease. We hypothesize that some unknown inflammatory trigger that is present in humans (and not present in mice) may be the necessary trigger for the development of PSEK. We further hypothesize that this stimulus may resolve spontaneously as children get older, leading to the eventual disappearance of the PSEK skin disease in older patients. While we have not identified the exact trigger in human PSEK, it is worthwhile noting that several common skin diseases, including atopic dermatitis and acne, that have complex interactions with skin microbiota, occur in younger individuals and tend to resolve in adults.

When subjected to IMQ topical application, a standard model of human psoriasis, Het I1029M mice showed exacerbated inflammation compared to WT (**Figure 2A**). This inflammation was characterized by higher mRNA expression levels of *Il17a* and accumulation of CCR6^+^ γδ T cells that is reported to be a major source of IL-17A in this model (**Figures 2D, E**). Then, we sought to investigate what cells are affected and contribute to the exacerbated inflammation in Het I1029M mice. Among the inflammatory cells, DCs are known to have a pivotal role in psoriasis. Importantly, TRPM4 is reported to be essential for DC migration (13). Our migration assay results clearly demonstrated that DC migration is enhanced in Het I1029M mice. In summary, the TRPM4 mutation may have two independent impacts on the exacerbation of PsD *via* increased proliferation of keratinocytes and enhanced migration of DCs. In Het I1029M mice, DC migration to the regional LN is enhanced (**Figure 3A**). These migrated DCs potentially stimulate LN T cells leading to expand IL-17A producing γδ T cells. As a result, increased accumulation of IL-17A producing γδ T cells in the lesions could lead to IL-17A-mediated dermatitis. Because keratinocytes are not the predominant cells that directly respond to IMQ, other cell types such as DCs expressing TLR7 should be activated by IMQ, resulting in the production of proinflammatory cytokines that then secondarily activate keratinocytes that express TRPM4. Recently in microglia, TRPM4-mediated NLRP3 inflammasome activation was reported (33). It is possible that TRPM4 GoF may mediate inflammasome-mediated signaling in the context of skin inflammation although this should be tested in future studies.

Interestingly, exaggerated inflammation in Het I1029M mice was only observed in IMQ-induced psoriasiform dermatitis model, but not observed in the croton oil-induced or DNFB-induced dermatitis models. Our results at least showed that DC migration and keratinocyte proliferation are associated with TRPM4 GoF. We speculate that diseases in which DC migration and keratinocyte proliferation play important roles would be greatly affected by TRPM4 GoF. As both DC migration and keratinocyte proliferation are the important features in psoriasis-like skin inflammation, that may explain why this GoF mutation specifically enhanced IMQ-induced skin inflammation. Clearly, other models of skin inflammation (such as tape-stripping) could be tested in the future to examine the specificity of TRPM4-mediated signaling.

Consequently, an important question is whether or not this exacerbated PsD in Het I1029M mice or spontaneous PsD in WT mice can be ameliorated by a TRPM4 inhibitor. TRPM4 channels can exist as homomers or assemble with sulfonylurea receptors (SURs) as complexes (34, 35). Glibenclamide inhibits SUR-TRPM4 heteromeric channels (34), but it may also directly inhibit TRPM4 channels (36, 37). Glibenclamide clearly ameliorated PsD both in Het I1029M and WT (**Figures 4A–D**, **5**). Moreover, glibenclamide clearly inhibited DC migration to the regional LN (**Figure 6**). These results help to re-affirm that the enhanced PsD observed in Het mice is a direct consequence of the TRPM4 channel. Since both WT and Het TRPM4 mice had reduced PsD when treated with glibenclamide, we suggest that TRPM4 may be a new and relevant target for psoriasis in humans.

There are several limitations in this study. One of the limitations is some of the results showed trends, but did not reach statistical significance due to the lack of availability of larger numbers of mutant mice. However, we note that these trends were observed in multiple experiments. Furthermore, we used only glibenclamide as a TRPM4 inhibitor because it is most widely used TRPM4 inhibitor used *in vivo*. Future studies using TRPM4 inhibitors other than glibenclamide are warranted. Lastly, while we were able to study keratinocytes and dendritic cells in greater detail, one limitation of this study is that we were not able to assess the biochemical characteristics of other immune cells such as T cells that might also be impacted by TRPM4 function. Because of multiple known functions of TRPM4 in different cell types, it is possible that TRPM4 GoF mutation regulates PsD in several ways, but, of note, a TRPM4 inhibitor blocks PsD in WT as well as TRPM4 GoF mice. Overall, these data indicate that TRPM4 may contribute to PsD in humans and that it warrants further study as a target in human psoriasis.

## Data availability statement

The original contributions presented in the study are included in the article/**Supplementary Materials**, further inquiries can be directed to the corresponding author/s.

## Ethics statement

The animal study was reviewed and approved by Institutional Animal Care and Use Committee, University of California, Davis.

## Author contributions

DY, SV, JZ, and SH designed the experiments, analyzed the data and wrote the manuscript. XW, ZS, and MH acquired, analyzed and interpreted the data. DM and JB analyzed and interpreted the data. All authors contributed to the article and approved the submitted version.

## Funding

Research reported in this publication was supported by the National Institutes of Health under award number 1R01NS128180-01 (to JZ) and Department of Dermatology Seed Grant (to SH).

## Conflict of interest

The authors declare that the research was conducted in the absence of any commercial or financial relationships that could be construed as a potential conflict of interest.

## Publisher’s note

All claims expressed in this article are solely those of the authors and do not necessarily represent those of their affiliated organizations, or those of the publisher, the editors and the reviewers. Any product that may be evaluated in this article, or claim that may be made by its manufacturer, is not guaranteed or endorsed by the publisher.

## Abbreviations

TRPM4, transient receptor potential melastatin 4; GoF, gain-of-function; PSEK, progressive symmetric erythrokeratodermia; CRISPR/Cas9, clustered regularly interspaced short palindromic repeats and CRISPR-associated protein 9; IMQ, imiquimod; PsD, psoriasiform dermatitis; IL, interleukin; DC, dendritic cell; EAE, experimental autoimmune encephalomyelitis; WT, wild-type; gRNA, guide RNA; HDR, homology directed repair; p-STAT3, phosphorylated signal transducer and activator of transcription 3; SUR, sulfonylurea receptor; IHC, Immunohistochemistry; H&E, hematoxylin and eosin; γδ-low, TCR γδ-low expressing; Th, T helper; CCR6, CC chemokine receptor-6.
