# Supplementary figures and images for: Gain-of-function of TRPM4 predisposes mice to psoriasiform dermatitis
Source: Front Immunol. 2022 Oct 20;13:1025499. doi: 10.3389/fimmu.2022.1025499 (PMC9632438; doi:10.3389/fimmu.2022.1025499)

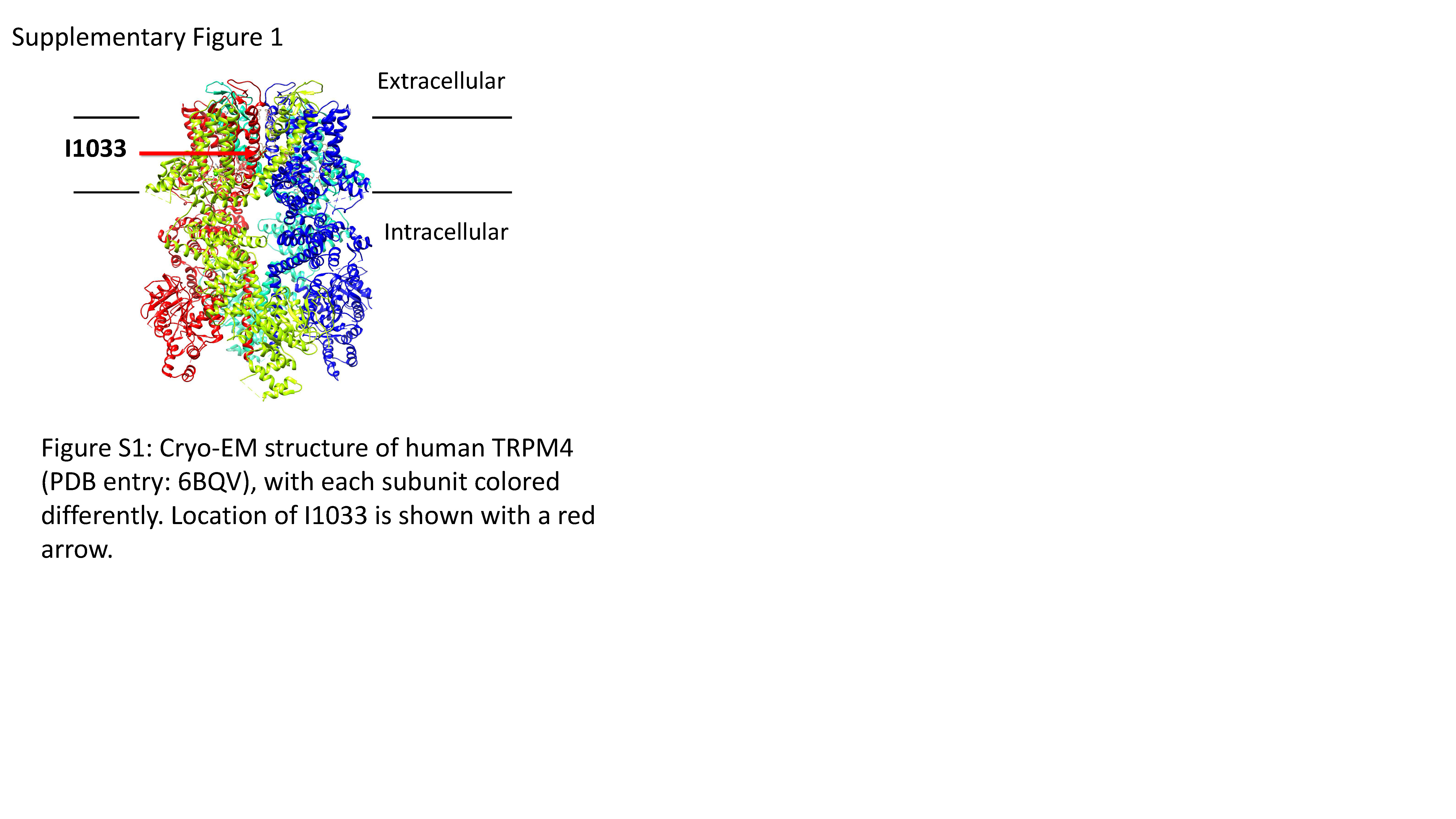

Supplement: Supplementary file 1 [file Image_1.jpeg]

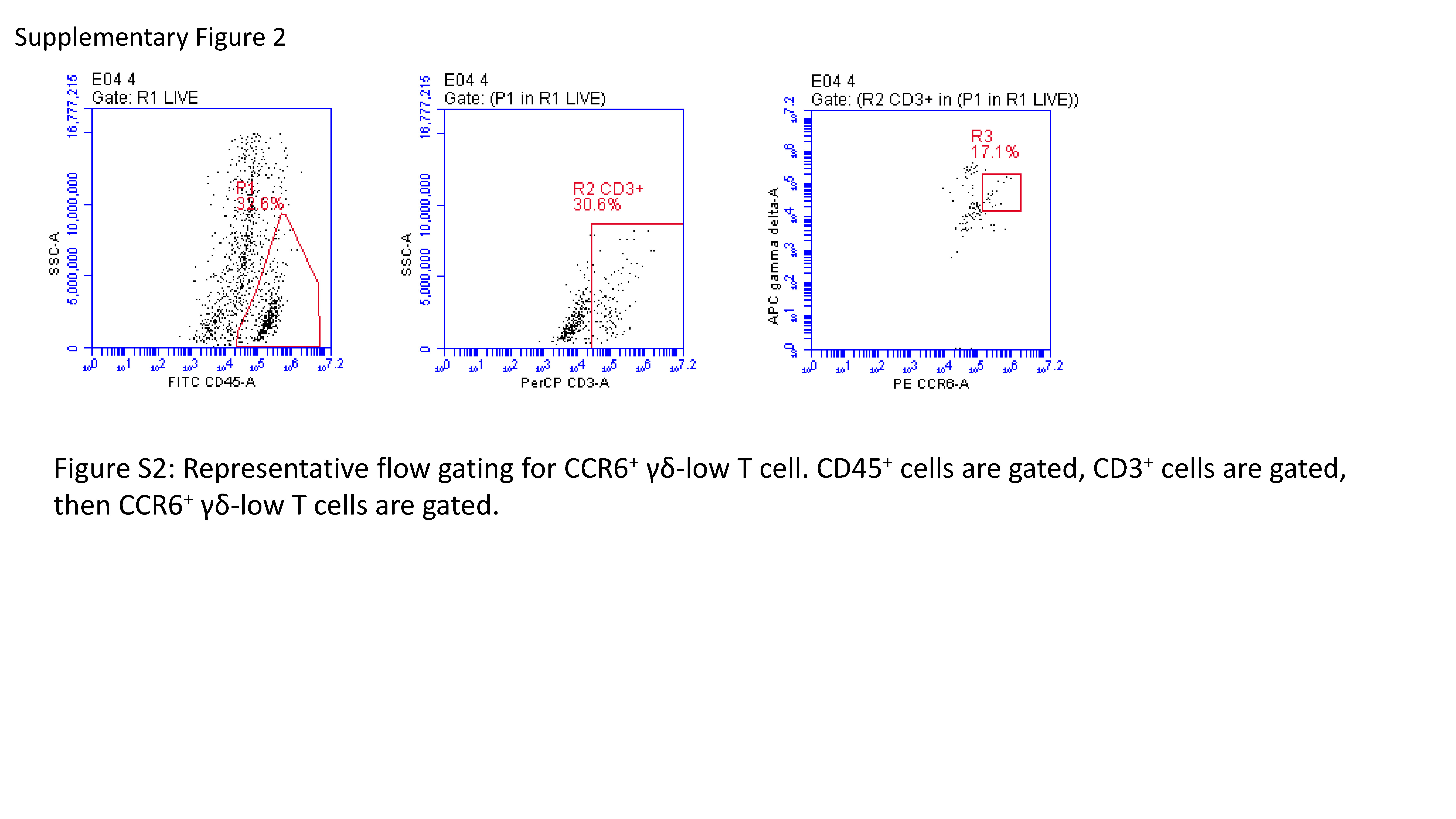

Supplement: Supplementary file 2 [file Image_2.jpeg]

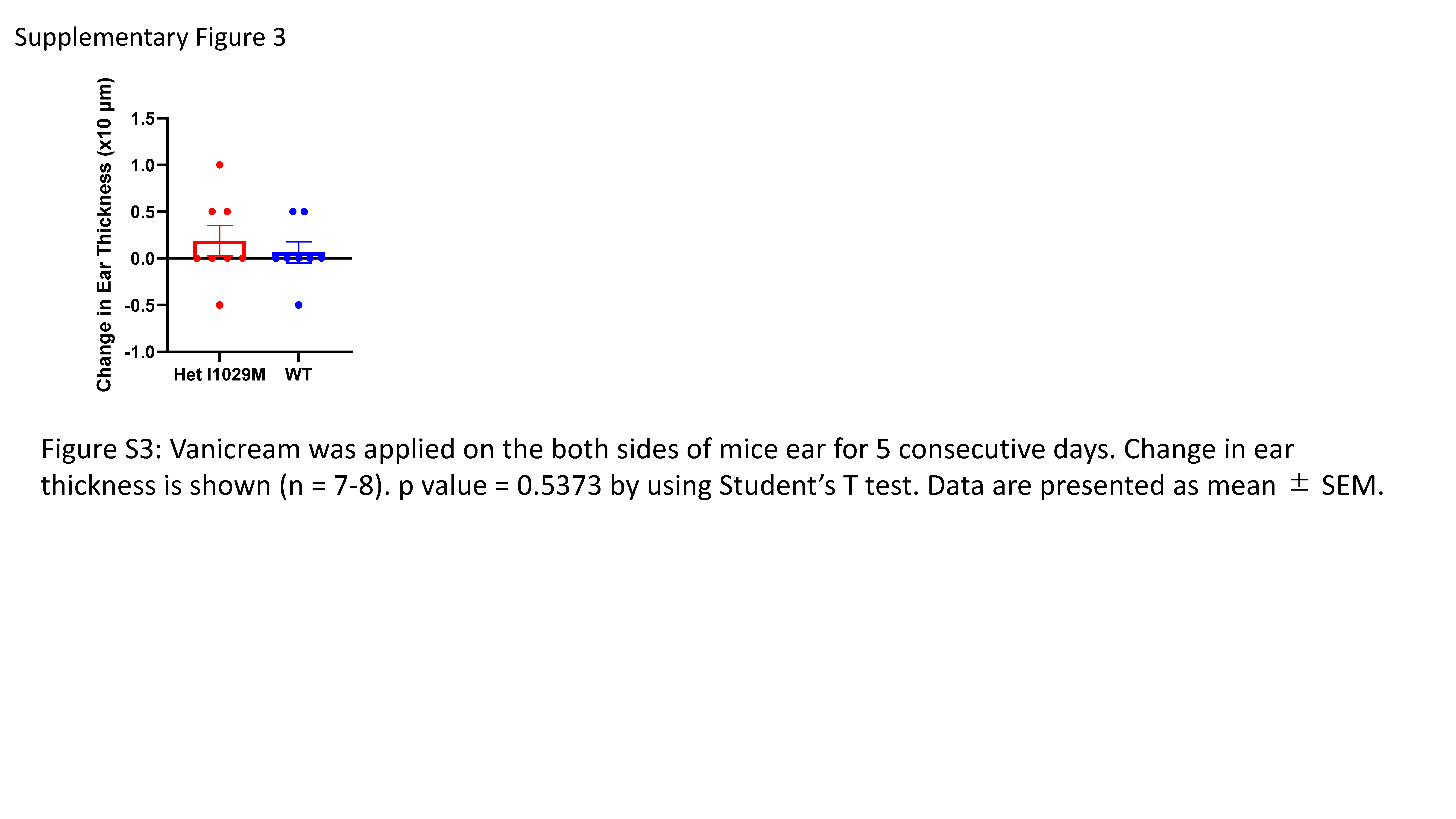

Supplement: Supplementary file 3 [file Image_3.jpg]

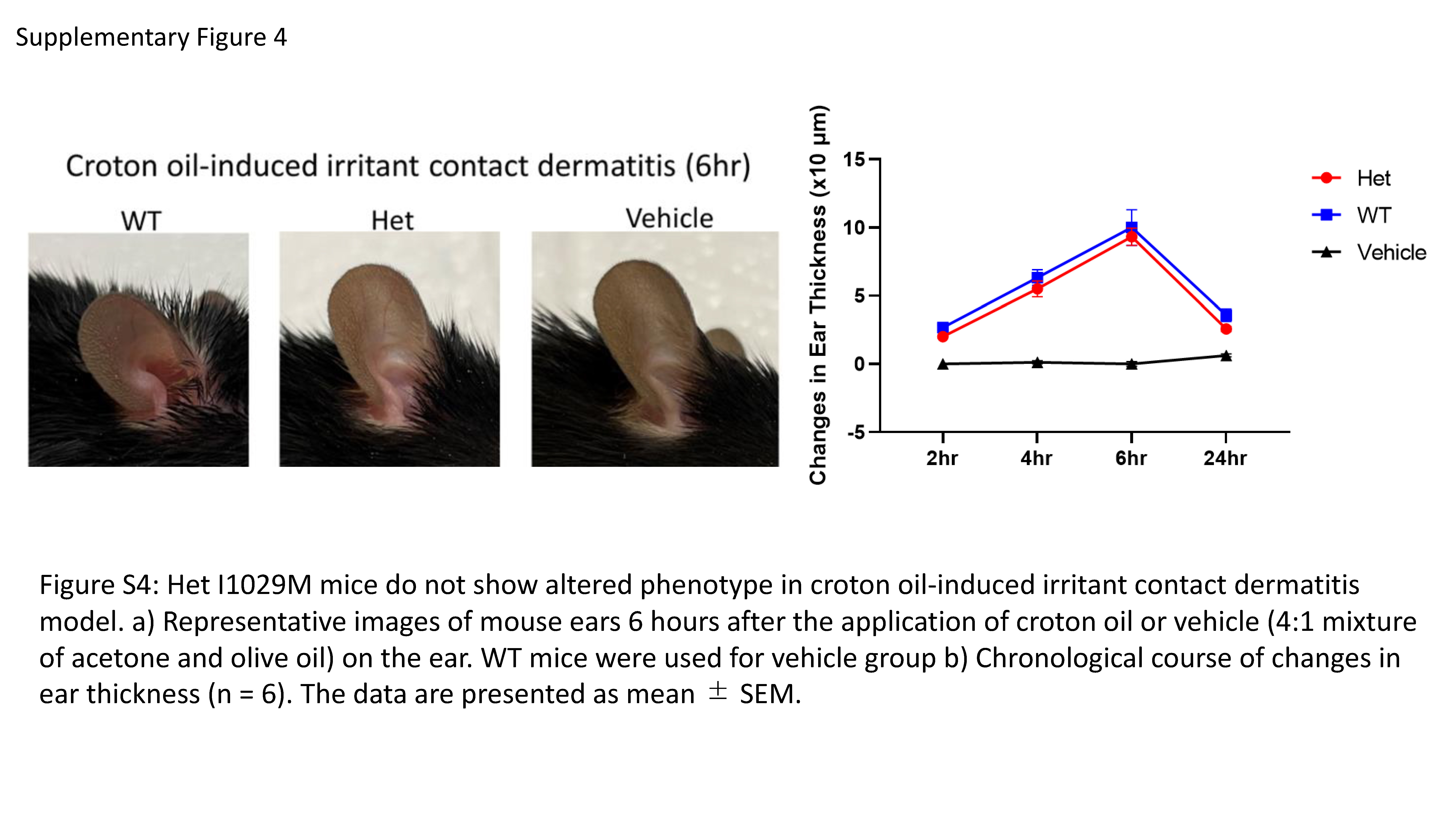

Supplement: Supplementary file 4 [file Image_4.jpg]

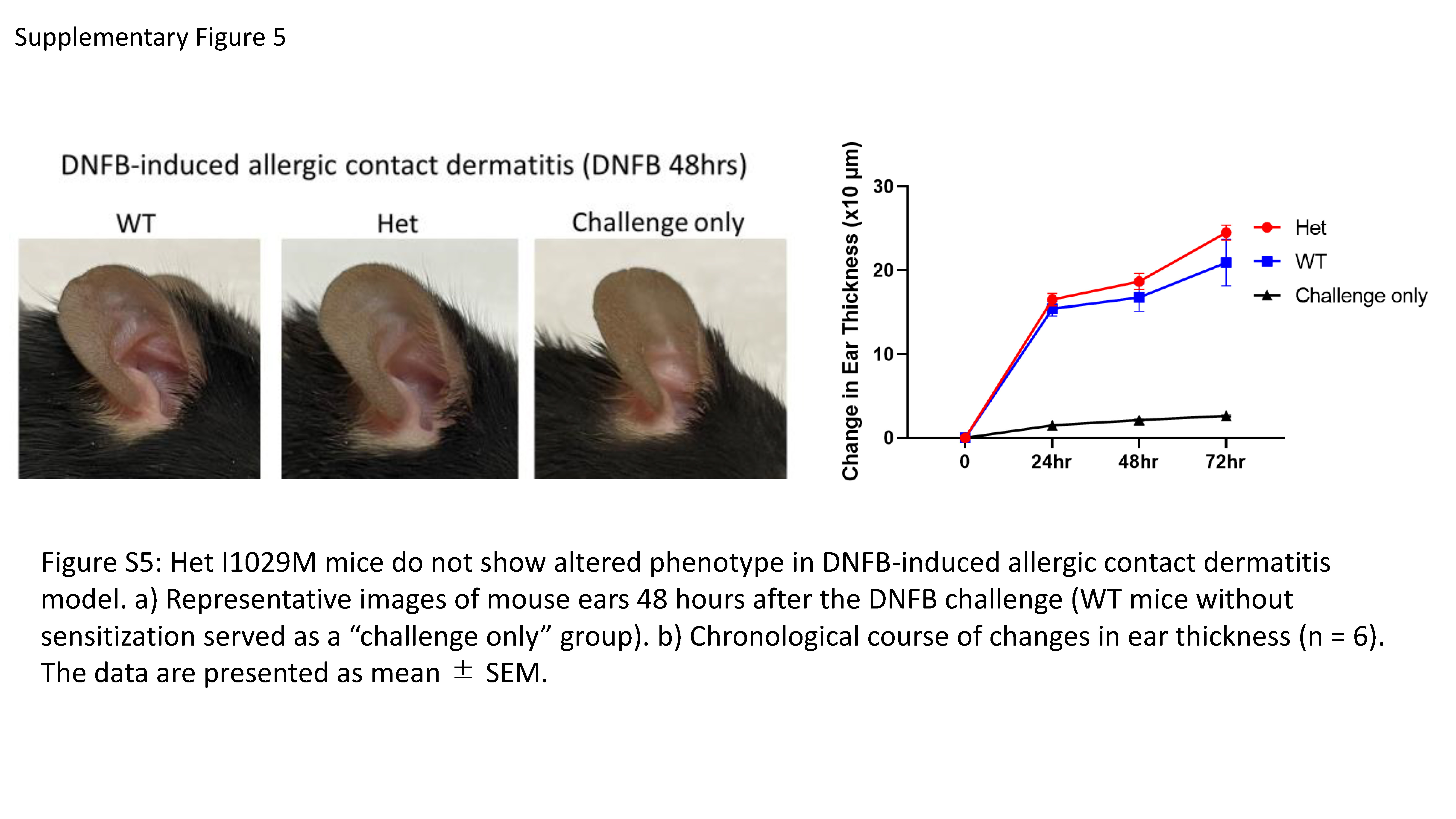

Supplement: Supplementary file 5 [file Image_5.jpg]

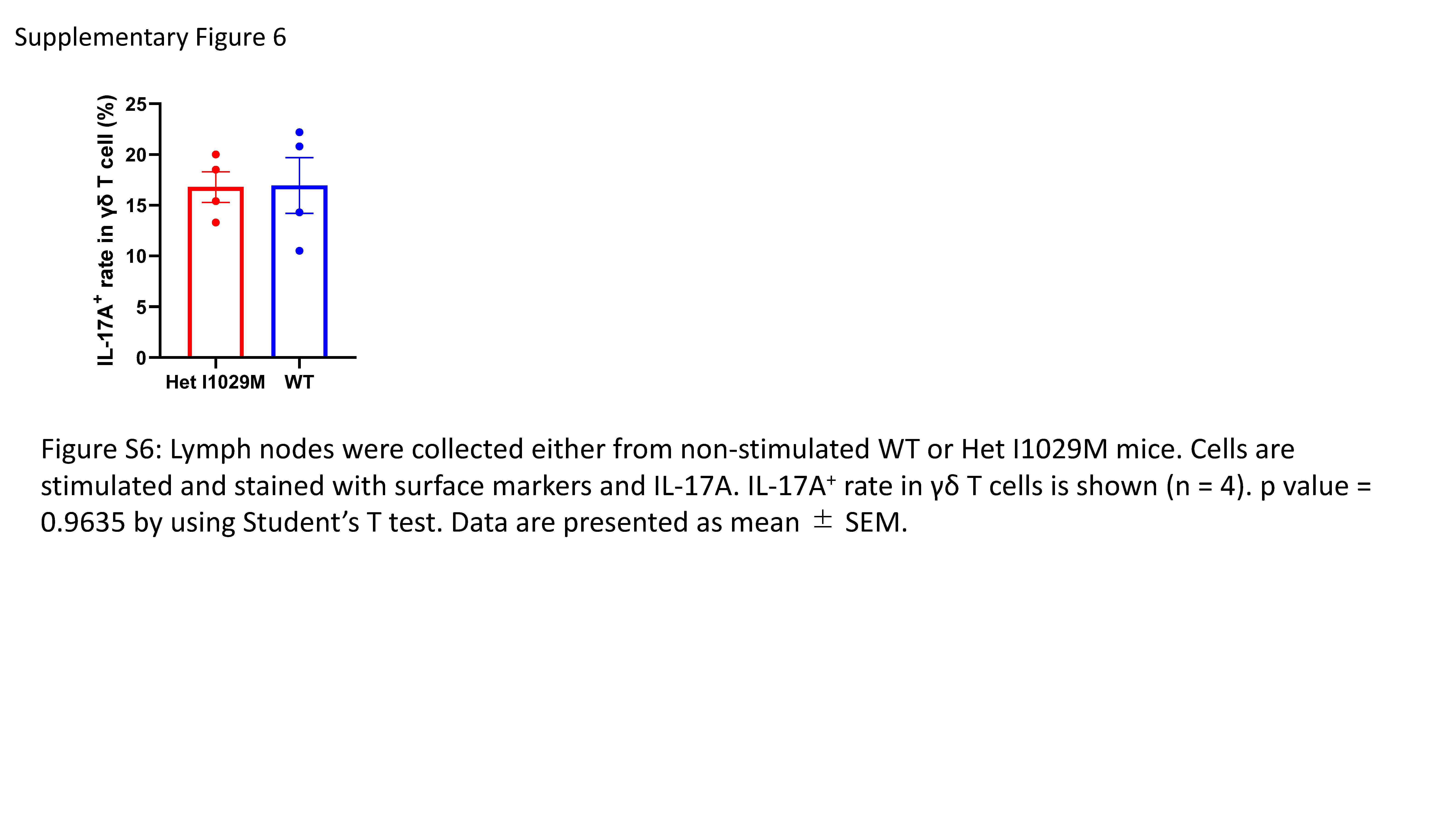

Supplement: Supplementary file 6 [file Image_6.jpg]
